# Revision and 90-day mortality following hip arthroplasty in patients with inflammatory arthritis and ankylosing spondylitis enrolled in the National Joint Registry for England and Wales

**DOI:** 10.1177/1120700021990592

**Published:** 2021-02-18

**Authors:** Laura L Miller, Daniel Prieto-Alhambra, Lea Trela-Larsen, J Mark Wilkinson, Emma M Clark, Ashley W Blom, Alexander J MacGregor

**Affiliations:** 1 Musculoskeletal Research Unit, School of Clinical Sciences, University of Bristol, Bristol, UK; 2 Musculoskeletal Pharmaco- and Device Epidemiology, Centre for Statistics in Medicine, Nuffield Department of Orthopaedics, Rheumatology, and Musculoskeletal Sciences, University of Oxford, Oxford, UK; 3 Department of Oncology and Metabolism, University of Sheffield, Sheffield, UK; 4 Norwich Medical School, University of East Anglia, Norwich, UK

**Keywords:** Ankylosing spondylitis, hip arthroplasty, mortality, osteoarthritis, revision, rheumatoid arthritis

## Abstract

**Aim:**

To assess revision rates and postoperative mortality in patients undergoing hip arthroplasty (HA) for inflammatory arthritis compared to hip osteoarthritis (OA).

**Methods:**

The analysis was conducted among cases of HA that were recorded in the National Joint Registry for England and Wales (NJR) between April 2003 and December 2012 and linked to Office for National Statistics mortality records. Procedures were identified where the indication for surgery was listed as seropositive rheumatoid arthritis (RA), ankylosing spondylitis (AS), other inflammatory arthritis (otherIA), or OA. 5-year revision risk and 90-day postoperative mortality according to indication were compared using Cox regression models adjusted for age, sex, American Society of Anaesthesiologists (ASA) grade, year of operation, implant type, and surgical approach.

**Results:**

The cohort included 1457 HA procedures conducted for RA, 615 for AS, 1000 for otherIA, and 183,108 for OA. When compared with OA, there was no increased revision risk for any form of inflammatory arthritis (adjusted HRs: RA: 0.93 (0.64–1.35); AS: 1.14 (0.73–1.79); otherIA: 1.08 (0.73–1.59)). Postoperative 90-day mortality was increased for RA when compared with OA (adjusted HR: 2.86 (1.68–4.88)), but not for AS (adjusted HR: 1.56 (0.59–4.18)) or otherIA (adjusted HR: 0.64 (0.16–2.55)).

**Conclusions:**

The revision risk in HA performed for all types of inflammatory arthritis is similar to that for HA performed for OA. The 3-fold increased risk of 90-day mortality in patients with RA compared with OA highlights the need for active management of associated comorbidities in RA patients during the perioperative period.

## Background

A substantial body of evidence supports the safety and effectiveness of hip arthroplasty (HA) in the management of end-stage osteoarthritis (OA).^1^ HA is also the recommended treatment for individuals with end-stage joint damage due to an underlying diagnosis of inflammatory arthritis.^2,3^ Reports have suggested that inflammatory arthritis can result in poor function postoperatively.^4^ The risk of revision has also been reported to be higher than in OA, although results have been conflicting with recent meta-analysis indicating that the increased risk of revision in inflammatory arthritis was confined to the first 3 months postoperatively.^5^ The rate of serious systemic infection is consistently reported to be higher in patients undergoing HA with rheumatoid arthritis (RA),^6,7^ as is prolonged hospitalisation.^8^ This increased complication rate following primary procedures has not been consistently associated with an increase in mortality,^5,8^ but substantial uncertainty remains.

Existing studies of the outcome of surgery in inflammatory arthritis have been limited by their methods of sample selection and relatively low sample sizes that have captured only few relevant outcomes for rheumatic disease. Reports have also focused on RA and often used a loose definition of disease that encompasses all types of inflammatory arthritis. The risks in ankylosing spondylitis (AS) and the seronegative inflammatory arthropathies have not been considered separately. These are distinct diseases: patients with different types of inflammatory arthritis are likely to be exposed to different degrees of systemic inflammation and treatment regimens and have different patterns of bone loss.^9,10^ All these factors have the potential to have a differential impact on the rate of complications following surgery, the risk of revision and on mortality.

The National Joint Registry (NJR) has been recording data on elective arthroplasty in England and Wales since 2003.^11^ The dataset of is a sufficient size to explore surgical outcomes in relatively uncommon subgroups of patients.

In this analysis, we assess the risk of revision following HA in patients with a recorded diagnosis of inflammatory arthritis and examine their mortality in the 90-day period following the procedure.

## Methods

### Data sources

The NJR was established in 2003 and captures all HA procedures including revisions, which can be linked within the register to the primary procedure. The NJR dataset also is routinely-linked to mortality data from the Office for National Statistics (ONS).

### Study population

Our initial dataset included data from linkable primary HA procedures performed on consenting patients in England and Wales between 1 April 2003 and 31 December 2012 for whom a valid patient-level identifier was available. Cases of revision were only included if a corresponding primary procedure could be identified within the NJR dataset.

### Classification of exposure

The NJR records the indications for surgery for all procedures using a data entry format that allows multiple indications to be listed for individual case. Data on inflammatory arthritis as an indication for surgery were initially recorded on the NJR as a separate data field in the core data set. This allowed us to classify inflammatory arthritis exposure categories as: (1) seropositive rheumatoid arthritis (RA); (2) ankylosing spondylitis (AS); and (3) other inflammatory arthritis (otherIA) (which was defined as seronegative rheumatoid arthritis, psoriatic arthritis and other unspecified inflammatory arthritis). A later version of the core dataset used for data collection introduced in December 2007 (and adopted gradually over subsequent years) did not distinguish between these different categories, hence the comparisons of outcomes among different types of inflammatory arthritis mainly relates to patients who had their primary surgery before 2007.

All 3 groups of inflammatory arthritis were classified regardless of their OA status. For the purpose of analysis, the comparison group included cases of OA where inflammatory arthritis had not been listed as a reason for surgery.

Procedures were excluded where OA or inflammatory arthritis was not recorded as an indication for surgery; where the data field relating to indication for surgery had not been completed; where any other indications for surgery were recorded other than avascular necrosis; where no data had been completed on the implant type; and where surgery had been carried out for trauma. Subjects undergoing simultaneous bilateral HA were excluded from the analysis of postoperative mortality. The data relate to the patients’ first recorded HA procedure: a small number of patients will have had a contralateral procedure prior to the start of the registry in 2003, but the exact numbers of these patients is not available.

### Study outcomes

The study outcomes were:

1. The 5-year revision risk, defined as revision surgery for any indication up to 5 years following primary HR and identified through record linkage within the NJR dataset.
2. The postoperative (90-day) mortality, defined as all-cause 90-day postoperative mortality based on record linkage to data from ONS.

The choice of the timing of end points was based on that standard practice of reporting for revision at intervals of 1, 3, 5, 7 or 10 years: we chose the longest time point where we would have sufficient data to allow a meaningful analysis. Excess postoperative mortality has been shown to occur in the first 90-days after hip replacement.^12^

### Statistical analysis

The 5-year implant survival and 90-day postoperative mortality for all 3 groups of inflammatory arthritis were compared with OA in a Cox proportional hazards regression models. Initially we considered minimally adjusted models, adjusted only for age and sex. We then compared these to adjusted models, which included American Society of Anesthesiologists (ASA) grade, year of operation, implant type (cemented or uncemented), and surgical approach (anterolateral, anterior, posterior or minimally invasive).

The 90-day mortality analyses also included thromboprophylaxis (chemical or mechanical) and anaesthetic type (general or spinal) in the adjusted models. All results are presented as hazard ratios (HR) with 95% confidence intervals (95% CI) and *p-*values.

The variables included in the regression analysis have been recognised to influence revision risk and mortality following HA. Prosthesis fixation method (cemented versus cementless) is also recognised to influence revision rates for OA.^11^ We investigated if the effect of fixation method on the risk of revision varied for each of the different inflammatory arthritis groups, by testing for the inclusion of interaction terms in the multivariable adjusted models using likelihood ratio tests.

In sensitivity analyses we examined if further adjustment for body mass index (BMI) affected the results. We used multiple imputation with chained equations to account for missing BMI data.^13^ As BMI was not collected in the NJR before 1 April 2004, it was assumed that BMI was missing completely at random, and these procedures were excluded from the imputation analyses. Imputation analyses were conducted for data relating to primary procedures where BMI had been recorded and any missing values were assumed to be missing at random. We included all covariates in the fully adjusted analysis model, a variable to indicate revision, and the Nelson-Aalen cumulative hazard estimate in the imputation equations for BMI. A total of 1000 imputations were completed.

A competing risks survival analysis was also conducted with mortality as the competing event. Sub-Hazard ratios (SHR) were estimated for 5-year implant survival for RA compared to OA using the Fine and Gray method.^14^ The statistical analyses were carried out using Stata 14.^15^

## Results

### Characteristics of the sample

**Figure 1.**
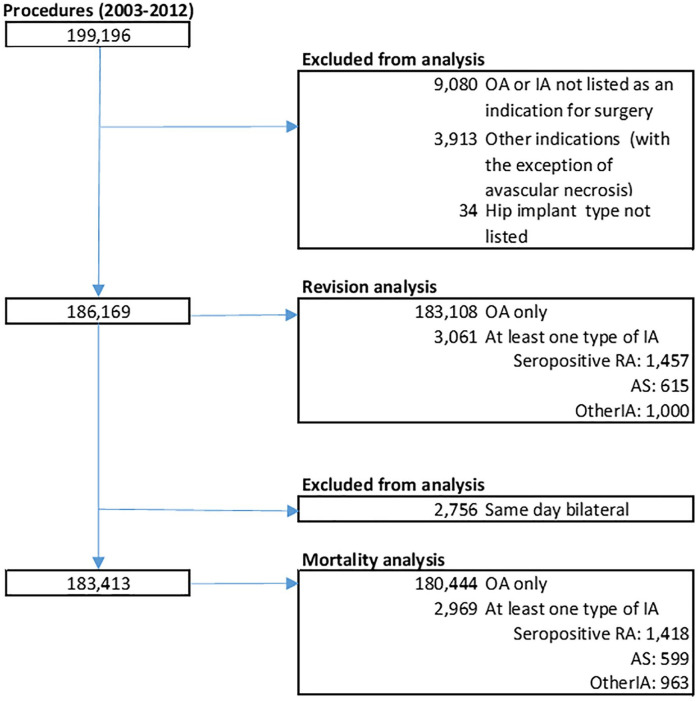
Population flow chart.

A total of 199,196 procedures were identified (Figure 1). We excluded 9080 procedures where OA or inflammatory arthritis was not recorded as an indication for surgery. We further excluded 3913 procedures performed for trauma or any other reasons except avascular necrosis. A total of 34 procedures were excluded where implant type was not recorded. For the mortality analysis, we excluded a further 2756 simultaneous bilateral procedures (1378 subjects).

A total of 186,169 procedures were included in the analysis of revision. Of these, Inflammatory arthritis was listed as an indication for surgery in 3061 (1.7%); RA: 1457, AS: 615, otherIA: 1000. OA (with no associated inflammatory arthritis) was listed as the indication for surgery in 183,108 (98.4%). There were 11 procedures in which >1 inflammatory arthritis indication had been included.

Compared with OA, HA procedures carried out for RA were more likely to involve cemented components (Table 1). Patients receiving hip operations because of inflammatory arthritis were younger, and had a greater level of comorbidity as indicated by an ASA grade 3 or above. Fewer resurfacing procedures were carried out for RA as an indication compared with OA. Those with RA and otherIA were more likely to be underweight than those with OA.

**Table 1.** Patient characteristics and implant type.

		OA		RA		AS		Other inflammatory arthropathy	
		*n*	%	*n*	%	*n*	%	*n*	%
All		183,108	98.4	1457	0.8	615	0.3	1000	0.0
Age (years)	<55	18,674	10.2	331	22.7	154	25.0	309	30.9
	55–59	17,905	9.8	202	13.9	72	11.7	130	13.0
	60–64	25,634	14.0	236	16.2	86	14.0	116	11.6
	65–69	32,059	17.5	247	17.0	93	15.1	144	14.4
	70–74	34751	19.0	206	14.1	75	12.2	123	12.3
	75–79	28,717	15.7	141	9.7	69	11.2	101	10.1
	⩾80	25,368	13.9	94	6.5	66	10.7	77	7.7
Gender	Female	108,771	59.4	1142	78.4	279	45.4	717	71.7
	Male	74,337	40.6	315	21.6	336	54.6	283	28.3
ASA grade	P1: Fit and healthy	43,913	24.0	125	8.6	114	18.5	172	17.2
	P2: Mild disease no incapacitating	115,746	63.2	772	53.0	381	62.0	574	57.4
	P3: Incapacitating systemic disease	22,443	12.3	538	36.9	114	18.5	237	23.7
	P4/5: Life threatening disease	1006	0.5	22	1.5	6	1.0	17	1.7
Hip type	Cemented	84,175	46.0	824	56.6	234	38.0	430	43.0
	Uncemented	51,664	28.2	315	21.6	207	33.7	297	29.7
	Hybrid	25,581	14.0	233	16.0	87	14.1	178	17.8
	Reverse Hybrid	2548	1.4	26	1.8	7	1.1	8	0.8
	Resurfacing	19,140	10.5	59	4.0	80	13.0	87	8.7
Surgical approach	Not posterior	88,867	54.4	704	56.3	313	50.9	468	52.6
	Posterior	74,442	45.6	547	43.7	302	49.1	421	47.4
Year of operation	2003	13,211	7.2	142	9.7	0	0.0	77	7.7
	2004	25,440	13.9	245	16.8	66	10.7	163	16.3
	2005	36,565	20.0	323	22.2	153	24.9	199	19.9
	2006	42,784	23.4	343	23.5	149	24.2	231	23.1
	2007	51,626	28.2	306	21.0	216	35.1	250	25.0
	2008	9726	5.3	74	5.1	21	3.4	57	5.7
	2009	2434	1.3	17	1.2	8	1.3	14	1.4
	2010	1151	0.6	5	0.3	2	0.3	8	0.8
	2011	171	0.1	2	0.1	0	0.0	1	0.1
BMI (kg/m^2^) category	Underweight (⩽18)	10,753	5.9	107	7.3	35	5.7	79	7.9
	Normal (19–25)	438	0.2	13	0.9	1	0.2	15	1.5
	Overweight (26–30)	12,550	6.9	71	4.9	32	5.2	57	5.7
	Obese (>30)	8311	4.5	33	2.3	20	3.3	30	3.0
	Missing BMI	151,056	82.5	1233	84.6	527	85.7	819	81.9

OA, osteoarthritis; RA, rheumatoid arthritis; AS, ankylosing spondylitis.

### Risk of revision

**Figure 2.**
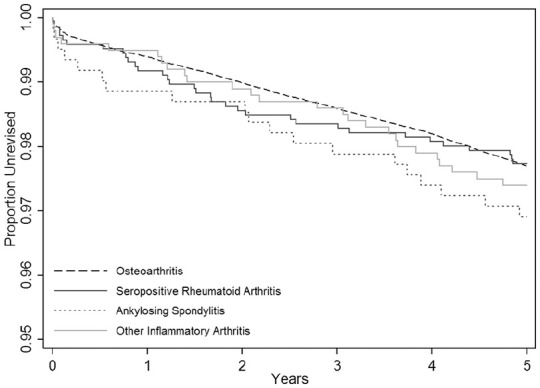
Implant survival according to type of arthritis.

The 5-year cumulative revision risk in those procedures undertaken for OA was 2.30% (95% CI, 2.23–2.37%). The unadjusted revision rates were all broadly similar to that for OA in all three inflammatory arthritis groups: (RA: 2.26% (95% CI, 1.62–3.17%), AS: 3.09% (95% CI, 1.98–4.80%) and otherIA 2.60% (95% CI, 1.78–3.80%)) (Figure 2). Table 2 shows the results of multivariate analysis. None of the models examined showed a significant difference in the hazard rate for revision for each of the three classifications of inflammatory arthritis compared with OA (adjusted model: RA: HR 0.93 (0.64–1.35; *p* = 0.70); AS: HR 1.14 (0.73–1.79; *p* = 0.57); otherIA: HR 1.08 (0.73, 1.60)).

**Table 2.** Hazard ratios for revision according to arthritis type (compared with OA).

Reason for surgery^a^	Adjusted for age and gender			Fully adjusted^b^		
	HR	95% CI	*p-*value	HR	95% CI	*p-*value
Seropositive RA (*n* = 1457)	0.796	0.565–1.122	0.193	0.929	0.64–1.351	0.701
Ankylosing spondylitis (*n* = 615)	1.078	0.687–1.693	0.743	1.141	0.727–1.792	0.566
Other inflammatory arthritis (*n* = 1000)	0.874	0.594–1.286	0.494	1.081	0.734–1.593	0.692

HR, hazard ratio; CI, confidence interval; OA, osteoarthritis; RA, rheumatoid arthritis.

a Includes procedures that also had OA recorded as a reason for surgery.

b Adjusted for gender, age, ASA physical status grade, hip replacement type, surgical approach and year of operation.

### 90-day mortality

**Figure 3.**
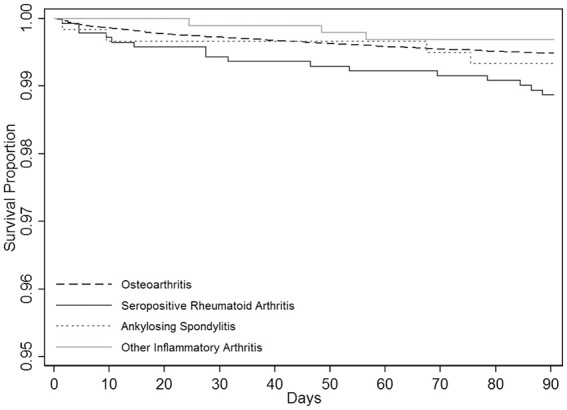
90-day patient survival according to type of arthritis.

The 90-day mortality rate in procedures carried out for OA was 0.52% (95% CI, 0.48–0.54%). Among the procedures carried out for inflammatory arthritis the mortality rate in RA (1.13% (95% CI, 0.67–1.79%)) was higher than in OA while the rates in AS (0.67% (95% CI, 0.24–1.72%)) and otherIA (0.30% (95% CI, 0.10–0.93%)) were broadly similar to the rate in OA (Figure 3). The increased 90-day mortality risk in RA was also reflected in the multivariate analysis (Table 3) where the age and gender-adjusted HR for RA compared with OA was 3.61 (2.20–5.92; *p* *<* 0.001)), and remained significant after further adjustment for ASA grade, hip implant type, surgical approach, year of operation, thromboprophylaxis (chemical or mechanical) and anaesthesia used. No significant differences in postoperative mortality when compared with OA were found for AS (adjusted HR 1.56 (0.59–4.18; *p* = 0.38)) and otherIA (adjusted HR 0.64 (0.16–2.55; *p* = 0.52)).

**Table 3.** 90-day mortality risk according to arthritis type (compared with OA).

Reason for surgery^a^	Adjusted for age and gender			Fully adjusted^b^		
	HR	95% CI	*p-*value	HR	95% CI	*p-*value
Seropositive RA (*n* = 1418)	3.605	2.195–5.919	<0.001	2.860	1.676–4.881	<0.001
Ankylosing spondylitis (*n* = 599)	1.587	0.594–4.240	0.357	1.563	0.585–4.180	0.373
Other inflammatory arthritis (*n* = 693)	0.960	0.309–2.984	0.943	0.637	0.159–2.554	0.524

HR, hazard ratio; CI, confidence interval; OA, osteoarthritis; RA, rheumatoid arthritis.

a Includes procedures that also had OA recorded as a reason for surgery.

b Adjusted for gender, age, ASA physical status grade, hip replacement type, year of operation, chemical thromboprophylaxis, mechanical thromboprophylaxis, and anaesthesia.

### Sensitivity analyses

#### Interactions

We found no evidence of an interaction between implant type and arthritis type on revision risk: *p*-values for interactions were 0.13 for RA, 0.26 for AS, and 0.47 for otherIA.

#### Multiple imputation and further adjustment for BMI in the analyses of revision risk in RA

BMI was missing for 82% of the procedures overall. Imputation based on 164,560 procedures where BMI data were assumed missing at random showed that additional adjustment for BMI resulted in a HR of for revision in RA compared with OA of 0.94 (0.64–1.36; *p* = 0.73). This compared to HR 0.93 (0.64–1.35; *p* = 0.70) in the original multivariable model, suggesting that BMI does not have a material influence on the results.

#### Competing risks in the analyses of revision risk in RA

When mortality was treated as a competing with the risk of revision, the HR for 5-year implant survival among procedures undertaken for RA compared with OA was 0.97 (0.66–1.41; *p* = 0.86). This was similar to the findings in models in which competing risks were not considered.

## Discussion

This national registry-based study shows that the risk of revision within 5 years of HA in patients with RA, AS or other types of inflammatory arthritis is similar to that seen in patients with OA alone. The findings contrast with the results of observational analyses that have suggested that the risk of revision is higher in patients with inflammatory arthritis and with the meta-analysis which showed an increased unadjusted risk of revision.^5^ The previous literature on the risks associated with joint replacement in RA attributed the increased risk of early revision to infection and dislocation.^6,16^ Our data suggest that, despite poorer short-term patient-reported outcomes,^17^ patients with RA undergoing joint replacement have similar implant revision rates as those with OA over the mid-term up to 5 years post procedure.

In contrast to the risk of revision, the 90-day mortality risk in patients undergoing these procedures enrolled on the NJR was not similar among these groups of patients. In patients with RA, while the overall risk of mortality following HA was low (1%), the 90-day mortality risk was almost threefold higher than in patients undergoing HA for OA alone. For AS and other types of inflammatory arthritis the mortality risk was similar to that observed in patients with OA alone. Patients with RA undergoing HA were younger than those with OA in our cohort and had a higher ASA grade than those with OA undergoing HA. The increased mortality risk in RA was independent of patient, implant, and surgical variables.

An increase in 90-day mortality was also seen among patients with inflammatory arthritis in an earlier analysis in the NJR dataset. Unlike the present study, the earlier analysis did not distinguish between the type of inflammatory arthritis.^12^ The observed increase in early postoperative mortality amongst patients with RA is supported by previous reports suggesting an increased risk of 90-day hospital readmissions related to infection (both local and systemic)^18^ and serious pulmonary events including need for mechanical ventilation.^8^ A systematic review of the literature suggests that the observed excess mortality might not appear immediately after surgery (in the first postoperative month) but rather in the following 2 months.^19^ However, our Kaplan Meier mortality data do not support this observation in this larger cohort.

Data on outcomes of joint replacement for individual types of inflammatory arthritis other that RA are sparse. A recent study on short-term outcomes following HA in AS has suggested that despite worse pre-surgery pain and function, 2-year patient-reported outcomes improved to equal those of patients undergoing similar surgery for OA.^20^ Our data are consistent with this, and showed no increased risk of either revision risk or 90-day postoperative mortality in AS patients compared to those undergoing HA for OA. The contrast in mortality between AS and RA may reflect the relative lack of comorbidity and general fitness in AS compared with RA, and may not have been fully accounted for in the multivariate model. Although no additional clinical data are available on these cases, it is likely that a smaller proportion of patients with AS will have been receiving disease modifying agents including biologics that may render them less susceptible to infective complications.

Our study has a number of limitations. The definition of arthritis type is a potential source for misclassification of exposure. It is however to be expected that surgeons would have coded this variable based on patient records, which should limit errors. The study outcomes (revision and death) cannot be validated at an individual level, although this should not affect the analysis of mortality, as date of death was obtained through record linkage. The cause of death could not be identified in the data available. Data on BMI were missing for a high proportion of patients. However, our sensitivity analysis found BMI had no impact on the observed associations. The problem of confounding by indication, where the choice of implant might be determined by patient characteristics, is also potentially limiting. We note, however, that adjustment for implant type had no material influence on our findings. While the confidence intervals for the estimates obtained for revision and mortality risks are relatively narrow, the findings also need to be tempered by power considerations. The study’s power also precluded us from addressing the influence of fixation type or individual reasons for revision.

The strengths of this study include the large sample size, and the length of follow-up available. Including potentially all patients and treatment centres in the NHS limits the potential for selection bias in previous cohorts. The findings are likely to be generalisable to Western European, Australasian and North American cohorts which exhibit similar demographics and follow similar treatment strategies.

## Conclusion

This is the first study to be conducted on sufficiently large scale to provide robust data on the rates of revision and postoperative mortality following HA in UK patients with different types of inflammatory arthritis. The findings will be of importance to patients and surgeons contemplating surgery for end stage disease of the hip. They also highlight the need to better understand the causes of the excess mortality risk in RA, and the impact of potential measures (specialised treatment centres or closer outpatient monitoring following discharge, amongst others) to minimise postoperative mortality amongst those undergoing HA for RA.
